# Preparation, Optimization and Evaluation of Chitosan-Based Avanafil Nanocomplex Utilizing Antioxidants for Enhanced Neuroprotective Effect on PC12 Cells

**DOI:** 10.3390/gels7030096

**Published:** 2021-07-16

**Authors:** Mallesh Kurakula, Raghavendra Naveen N., Bhaumik Patel, Ravi Manne, Devang B. Patel

**Affiliations:** 1 Department of Biomedical Engineering, The University of Memphis, Memphis, TN 38152, USA; 2 Department of Pharmaceutics, Sri Adichunchanagiri College of Pharmacy, Adichunchanagiri University, B.G.Nagar 571448, Karnataka, India; raghavendra.naveen@gmail.com; 3 Product Development Department, Cure Pharmaceutical Corporation, Los Angeles, CA 90025, USA; Bhaumikp17@gmail.com; 4 Chemtex Environmental Laboratory, Quality Control, and Assurance Department, Port Arthur, TX 77642, USA; ravimannemr@gmail.com; 5 Department of Pharmaceutical Sciences, Arnold and Marie Schwartz College of Pharmacy and Health Sciences, Long Island University, Brooklyn, NY 11201, USA; devang513@gmail.com

**Keywords:** avanafil, chitosan, alpha lipoic acid, ellagic acid, optimization, diabetic neuropathy

## Abstract

(1) Introduction: in recent decades, interdisciplinary research on the utilization of natural products as “active moiety carriers” was focused on due to their superior safety profile, biodegradability, biocompatibility and the ability for sustained or controlled release activity. The nano-based neuroprotective strategy is explored as an imperative treatment for diabetic neuropathy (DN). Avanafil (AV), that selectively inhibits the degradation of cGMP-specific phosphodiesterase, thereby increasing the levels of cGMP, makes a decisive mediator for cytoprotection. (2) Methods: AVnanocomplex formulations were prepared by a modified anti-solvent precipitation method and the method was optimized by Box–Behnken design. An optimized formulation was characterized and evaluated for various in vitro parameters; (3) results:based on the desirability approach, the formulation containing 2.176 g of chitosan, 7.984 g of zein and 90% *v/v* ethanol concentration can fulfill the prerequisites of optimum formulation (OB-AV-NC).OB-AV-NC was characterized and evaluated for various parameters. The neuroprotective mechanism of AV was evaluated by pretreatment of PC12 cells with plain AV, avanafil nanocomplex (NC) without antioxidants (AV-NC) and with antioxidants (α-Lipoic acid LP; Ellagic Acid EA), AV-LP-EA-Nanocomplex has also shown considerable attenuation in intracellular reactive oxygen species (ROS) and lipid peroxidation with a significant increase in the PC 12 viability under HG conditions in comparison to pure AV; (4) conclusion: the nanocomplex of AV prepared to utilize natural polymers and antioxidants aided for high solubility of AV and exhibited desired neuroprotective activity.This can be one of the promisingstrategy to translate the AV nanocomplex with safety and efficacy in treating DN.

## 1. Introduction

Avanafil (AV) is the second-generation phosphodiesterase-5 enzyme inhibitor (PDE-5), used as an effective medication for erectile dysfunction [1,2]. Erectile dysfunction is the inability of men to attain or maintain enough erection in completing the sexual activity [3] and as a public health issue; this can impact psychological condition, quality of life and the marital relationship of patients [4]. AV was first approved by the USA ood and Drug Administration (FDA) in 2012 followed by the European Medicines Agency in 2013 [5]. AV is more selective for PDE-5 and other cellular targets than sildenafil and vardenafil [6]. On the other hand, the poor aqueous solubility of AV (<1 mg/mL at 25 °C, in water, methanol and ethanol) contributes to the low bioavailability (38–41%) [7]. Hence, there is a need to enhance bioavailability by increasing the solubility of AV to make an effective formulation and this can be achieved by using nanotechnology, and polymer based nanocomplex is one of the advancing carriers among them.

Alpha-Lipoicacid (α-LP) is a potentantioxidant, which is essential for aerobic metabolism(Moon 2016). Ellagic acid (EA) is a dimeric subordinate of gallic acid and owing to its antioxidant property. EA has been reported for its role as anticarcinogenic and antiviral properties as well. Furthermore, EA can lessen lipid peroxidative makers by intensifying the antioxidant path. Both LP and EA are reported for synergetic antioxidant effects and additionally, they can cause higher entrapment of active moieties in nanoparticles and related dosage forms [8]. Zein is the major protein present in the maize, containing four main fractions such as α-, β-, γ- and δ-zein.It belongs to the prolamin class, accounting for about 50% of the total protein content. Nanostructured chitosan can inhibit several tumor establishments and have more retention time in blood. Chitosan has several reactive amino groups, and this exceptional characteristic nature can provide a high affinity towards biological site-specific targeting that can also offer interaction with many nanostructured materials to form a huge variety of composites. Nanoparticles or microparticles can be simply prepared by employing an ethanolic solution of zein.Nanocomplex formulation can increase the surface area, saturation solubility finally can enhance the drug release rate to provide the required concentration of drugs at the targeted site. Many of the drugs are unable to cross the neurobarrier in their original form, but this can be overcome by nanocomplex formulation aiding for better neuroprotection [9]. 

The impact of several neurodegenerative diseases (Parkinson’s disease, Alzheimer’s disease and Diabetic neuropathy, etc.,) is noticeably enlarged and at present, the pathogenesis of these disorders is not fully implicated thus none of the current treatments can impede their progression [10]. The efficiency of L-Dopa therapy declines on use for several years and besides, persistent treatment with L-Dopa may result in motor and non-motor adverse effects [11,12]. Fora few years, there is a remarkable interest in the use of PDE-5 inhibitors (PDEI) as therapeutic targets for these neurodegenerative diseases. PDEI can selectively inhibit the degradation of cGMP-specific phosphodiesterase, which normally terminates the cGMP signal through hydrolysis of cGMP to GMP [13,14]. A wide range of research papers confirmed the role of cGMP as a decisive intermediary of cytoprotection. Consequently, the use of PDEI leads to the build-up of cGMP concentration and enhances its performance [15,16].

PC12 cells are widely used as models for both neuroendocrine secretion and neuronal differentiation. Widespread use of PC12 cells is to study the neurotoxic activity of various substances, for example, by assessment of the effect on cell survival, neurite outgrowth, DNA damage or protein expression levels. However, this cell line is also widely used as a model for neurodegenerative diseases.

The present study plans to improve the AV solubility by using natural polymer-based nanocomplex augmented with natural antioxidants to repurpose its use in neuroprotection in diabetic neuropathy. The prepared nanocomplex was characterized, optimized using a mathematical tooland evaluated to identify the role of AV in antioxidant conjunction inaiding synergetic neuroprotective effect as a potential benefit in treating diabetic neuropathy.

## 2. Results

### 2.1. Thermal Analysis

DSC was used to evaluate the changes in the crystallinity and solid phase of the selected formulationsand their respective components. DSC thermograms of pure AV and optimized formulation (DSC thermogram enclosed in supporting documents).

### 2.2. Optimization of Chitosan-Avanafil Nanocomplex Formulation

Box–Behnken design withsurface response methodology was used to analyze the optimal levels of selected factors and their interactions resulting in minimum sizewiththehighest entrapment and stability index(Vyas et al. 2010).Here, 17 runs were anticipated, and the observed responses are given in Table 1.The particle size of all the trial formulations was found to be in the range of 109 to 355 nm, AV entrapment was about 46 to 87%, while stability was estimated in the range of 57 and 98%. All the obtained results were analyzed for individual responses and the effect of variables was studied statistically using the f_x_ model and ANOVA.

On basis of the sequential sum of squares (Type I) (F-value, *p*-value) and fit summary (Adjusted and predicted R^2^), the quadratic model was selected for all the responses [Table 2] [17].The quadratic model was selected (highest order polynomial) since the additional terms are significant and the model is not aliased.

**Figure 1:**
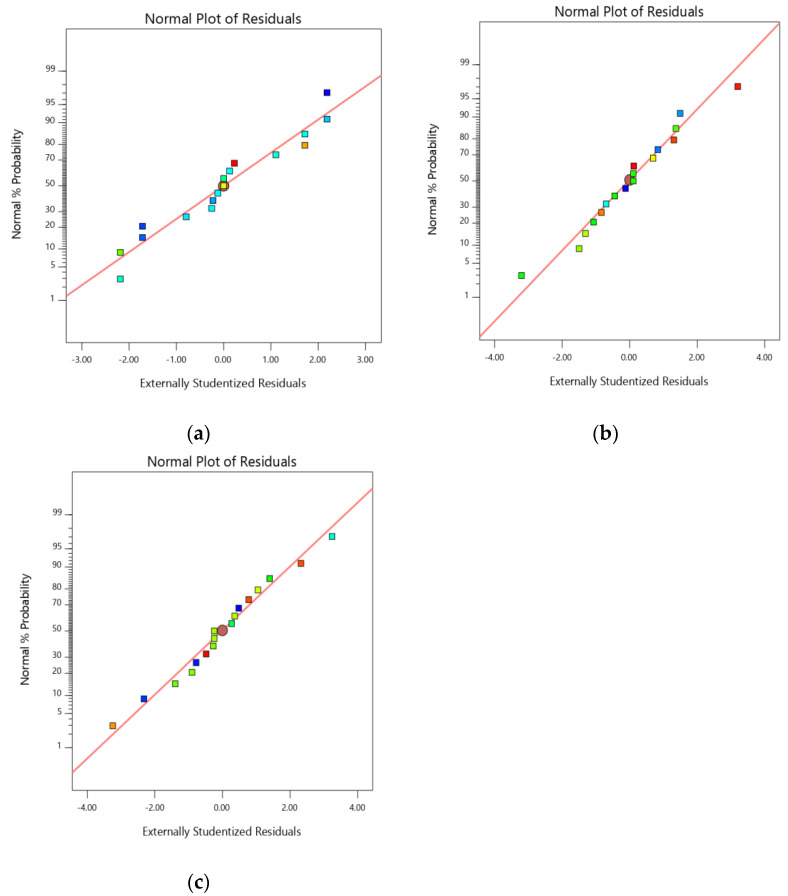
Normal probability plots of the residuals for (**a**) PS (**b**) EE and (**c**) SI.

**Figure 2:**
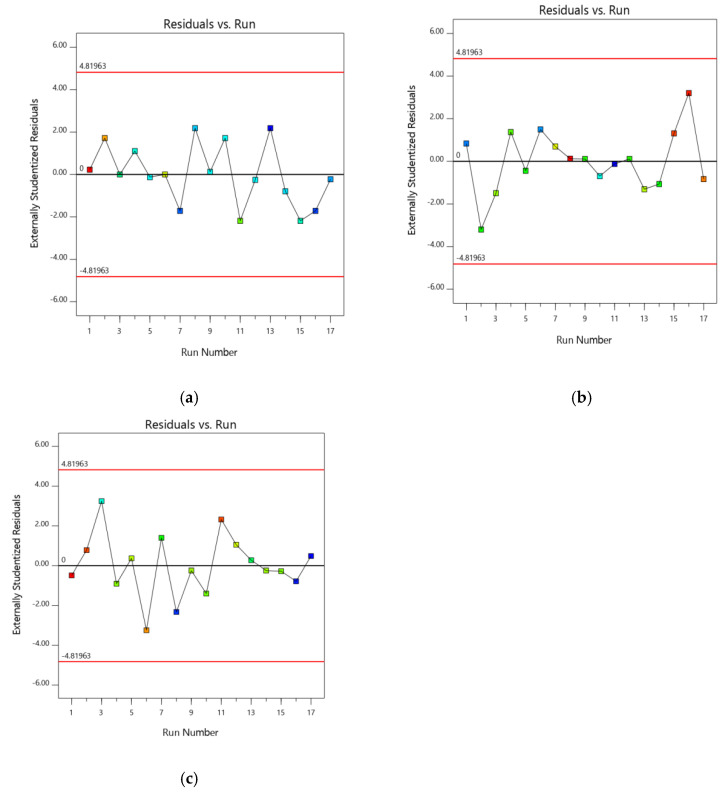
Model residuals Vs. test orders for (**a**) PS (**b**) EE and (**c**) SI.

A normal plot of residuals (Studentized residuals vs. normal% probability) was additionally used to quantifying the derived models to confirm the accuracy of the model (Figure 1 and Figure 2) [18]. In addition, the influence of test orders on the derived design was demonstrated by model residuals versus test orders [19]. ANOVA was performed to study the inference of the quantitative effects of the fact factors. Overall data were subjected to multiple regressions to yield polynomial equations.

**Figure 3:**
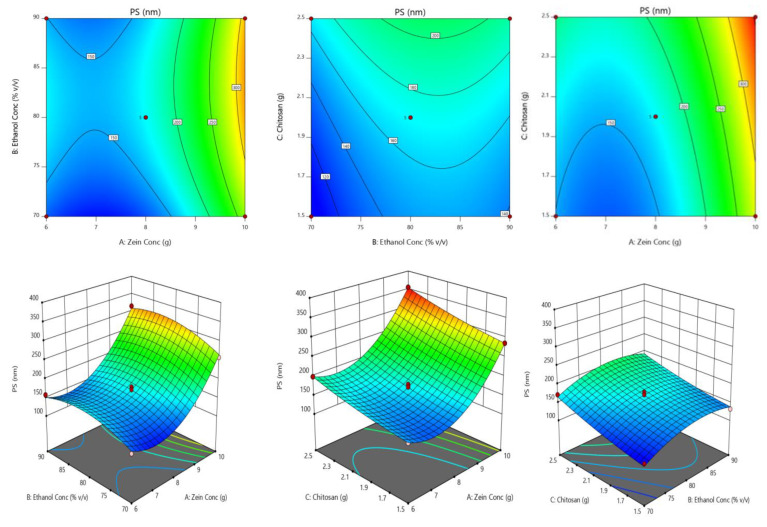
Response surface graphs for particle size (PS) (contour and three-dimensional).

Calculated F values, *p* values and lack of fit for selected responsesareshown in Table 3.These values were used to measure the significance of the coefficients of the model. The effect of independent variables on responses was further elucidated and analyzed by using RSM [20]. Figure 3 indicates the interaction of selectedresponses (particle size) with the variables, contour plot, which confirms the effect of variables. The derived equations of the responses for the best fit model were given as follows.

PS = +171.00 +73.00 A +15.75 B +29.50 C +6.00 AB +4.50 AC − 2.50 BC +70.25 A^2^− 27.25 B^2^ +5.75 C^2^ (1)

EE = +67.80 − 13.94 A +6.44 B − 2.38 C +5.00 AB +2.13 AC +6.38 BC − 2.40 A^2^ +6.10 B^2^− 1.02 C^2^ (2)

SI =+84.40 +17.12 A +0.6875 B +3.81 C − 0.2500 AB − 1.50 AC − 0.8750 BC − 3.89 A^2^− 3.76 B^2^− 1.51 C^2^ (3)

Global desirability function (D) was used to optimize different series of models that were obtained from the experimental analysis [21]. The combined desirability plot for all the responses has shown a maximum D value of 0.8964, which was obtained at optimum concentrations of independent variables. Based on this desirability approach, a formulation containing 7.984 g of zein, 90% *v/v* ethanol concentration and 2.176 g of chitosan can accomplish the prerequisites of the optimized formulation.

### 2.3. Zeta Potential and PDI

The polydispersity of the optimized formulation (OB-AV-NC) can be confirmed by a very low PDI of 0.14 ± 0.07. The surface charge was found to be +32.1 mV, which confirms the high stability of the prepared formulation.

### 2.4. Surface Morphology Studies

**Figure 4:**
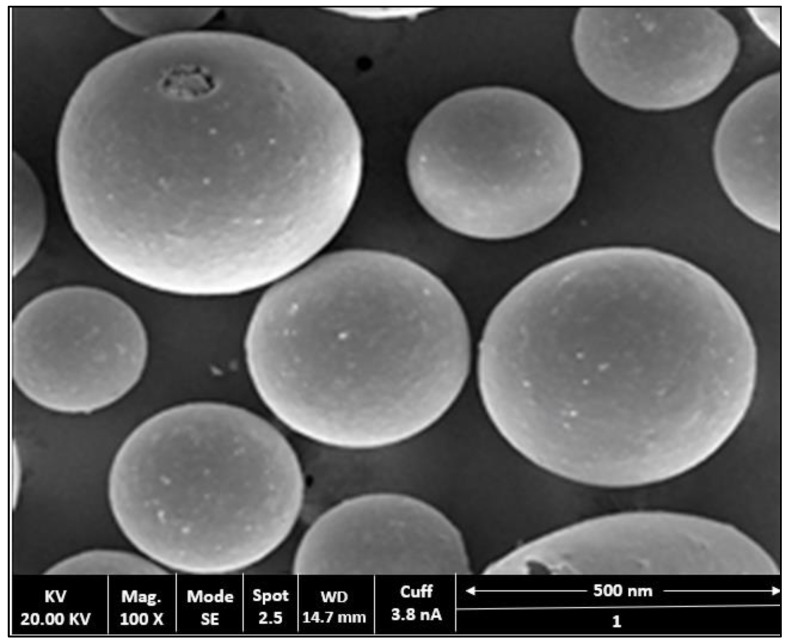
SEM image of optimized formulation (OB-AV-NC).

The microstructures of prepared AV-NC were characterized by SEM. As presented in Figure 4, the formulationswere recorded as small spheroid complexes havingslightly smooth to rough surfaces.

### 2.5. In-Vitro Release Study

**Figure 5:**
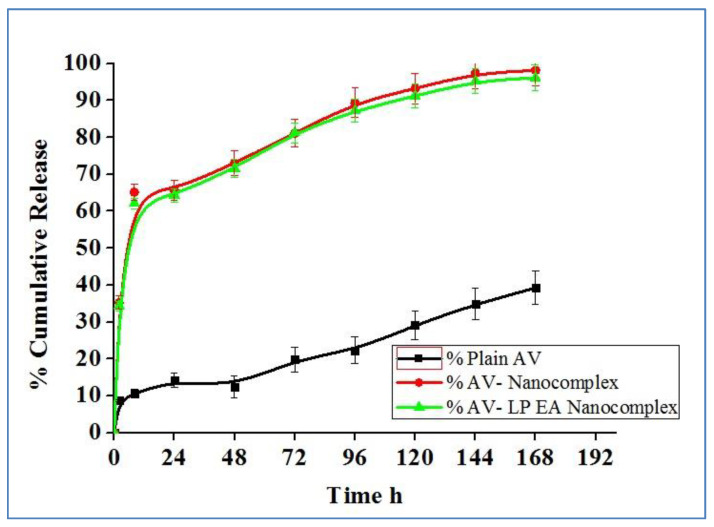
Comparative cumulative release profiles of plain AV, AV-NC and. AV-LP-EA-NC.

The cumulative release of AV from plain AV, AV-NC and AV-LP-EA-NC was determined and the data depicted in Figure 5. Drug release studies were performed up to 168 h. At 8 h, a peak release of AV was observed from both nanocomplex formulations, and thereafter steady-state release was observed up to 168 h. There were no marginal changes in the AV release from AV nanocomplex and AV LP EA nanocomplex.

### 2.6. Measurement of Cell Viability

**Figure 6:**
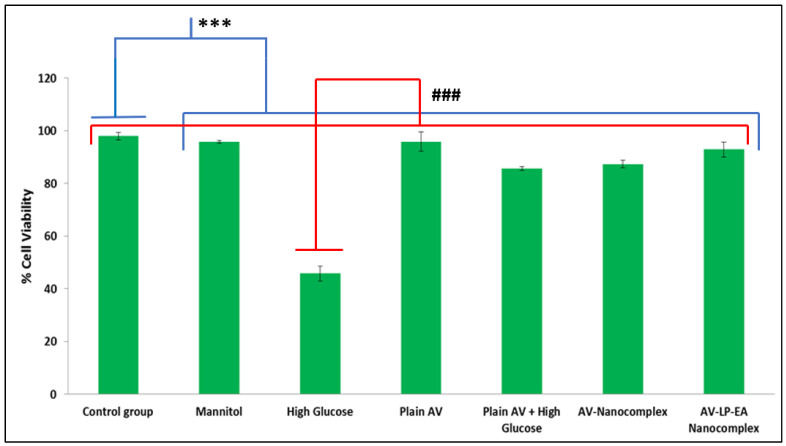
MTT assay shows cell viability of different groups with respect to control (Results are mean ± SD of threeindependent experimentsperformed in duplicate) The difference between control and other groups is significant *p* < 0.001 (***) and thedifference between high glucose and other groups is significant *p* < 0.001 (^###^).

The protective effect of AV on PC12 cells was determined by MTT assay. High glucose (HG) condition considerably decreased the cell viability after 72h, while pre-treatment with plain AV and AV-LP-EA-NC decreased the cell toxicity (Figure 6).

### 2.7. Measurement of Intracellular ROS

**Figure 7:**
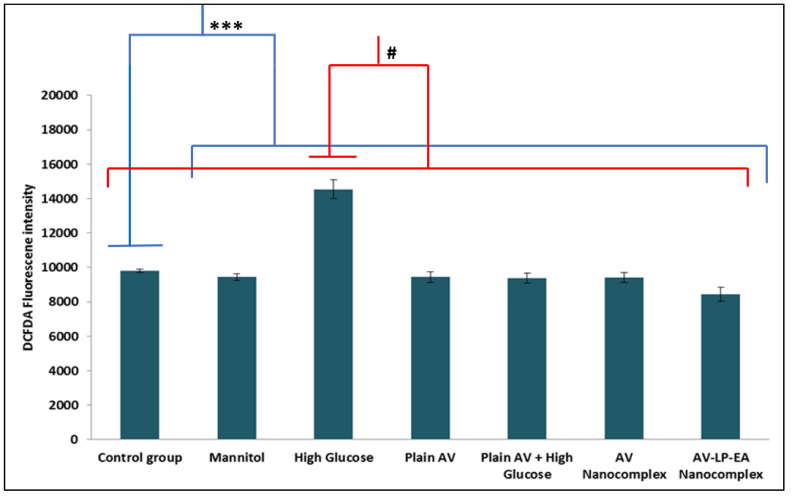
ROS levels measurement of different groups (Results are mean ± SD of threeindependent experiments) (***-The difference between control and other groups is significant *p* < 0.001 (***) and the difference between high glucose and other groups is significant *p* < 0.05 (^#^) Measurement of lipid peroxidation.

The anti-oxidant effect of AV was determined by measuring the intracellular ROS level. HG-treated cells showed maximum levels (14,533.62) of ROS, while pre-treatment with plain AV and NC formulations significantly reduced the level of ROS as shown in Figure 7.

**Figure 8:**
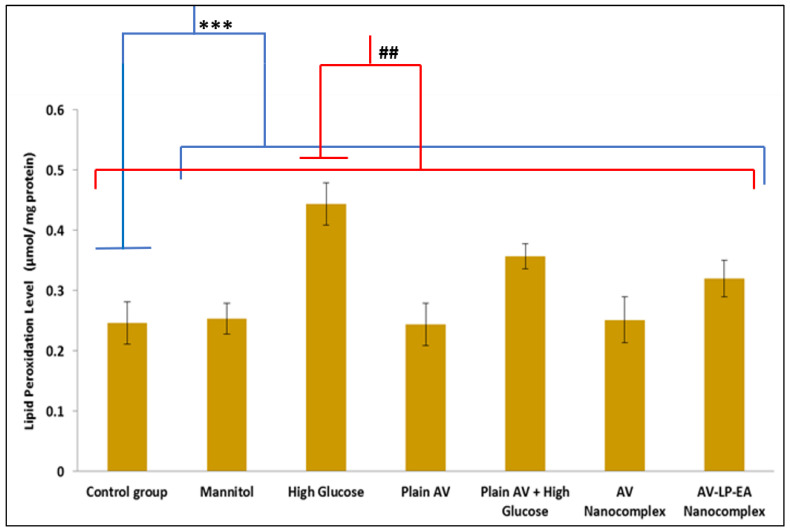
Lipid peroxidation measurement of different groups.Results are mean ± SD of three independent experiments performed in duplicate.The difference between control and other groups is significant *p* < 0.001 (***) and the difference between high glucose and other groups is significant *p* < 0.01 (^##^).

Pre-treatment of cells with formulation AV-LP-EA-NC noticeably attenuated the lipid peroxidation change to 27.27% compared to the HG condition (Figure 8). MDA levels in the control group, mannitol group, and AV pre-treated with PC12 cells under NG condition, were almost similar.

### 2.8. Discussion

AV showed a sharp endotherm at 162 °C, matching its melting point.DSC thermogram of optimized formulation had two melting endotherms, one at 72.5 °C and the second was noted at 163.2 °C. There was no such change in the melting point of AV, thus concludes the same state of AV in the formulation, without interacting with polymers [supplementray data]. The root effect interrelationship between chosen parameters and the individual response would be expressed by the suggested quadratic model and respective statistical significance wasdetermined by ANOVA. The quadratic model was chosenbasedonthesequential sum of squares (Type I), where all the existing models were calculated for F and *p*-value. The *p*-value for PS, EE and SI was found to be <0.0001, 0.0015 and 0.0015, respectively. The fit summary for the selected responses had shown anacceptablefit with experimental data, which is certified by inflated adjusted and predicted R^2^ values (0.9857, 0.9199 for PS; 0.9760, 0.8845 for EE, 0.9822, 0.9035 for SI). The data showed an <0.2 difference, implicating that the adjusted R^2^was in good agreement with the predicted R^2^ values. To estimate the signal to noise ratio (predicted response related associated error),i.e., ‘Adequate precision’is used. To traverse a design space the adequate precision ratio being above four is a prerequisite [22]. Further runs in determining the SI, PS and EE indicated a signal-to-noise ratio of 39.95, 27.92 and 31.80, proving the high acceptability of the chosen model. The coded factors associated with equating terms are useful in making better predictions over the response at all levels of individual factors. Each charter equation compares the factor coefficients that help in recognizing the relative impact of factors. A high interdependence was found with acquired experimental data and predicted data when representing two investigated responses graphically. The normal probability plot ensures that the residuals are followinga normal distribution (straight line by all points). Formal statistical tests are not used for this, and visual inspection of the graph is adequate. The promising metric to use in the plot is extrinsic studentized residuals whereas other raw or internal studentized residuals are not advocated.

In the current study, a straight-line distribution for the extrinsic studentized residuals with a little deviation (Figure 1) was observed indicating that the selected model was statisticallyaccepted [23]. Figure 2 represents experimental runs versus the residuals, in fact, an operational way of identifying the lurking variables affecting the responses during the experiment. A random scatter trend is observed in the plot indicating a time-coupled variable slink in the background. The reproducibility of the model can be confirmed with the CV value (coefficient of variation). The reproducibility of the current model has observed CV < 10%.

Comparatively lower CV values of 4.25% for PS; 2.76% for EE and2.14% for SI were recorded from this study, confirms the reliability and accuracy of the model. Lack of fit can measure the inability of the model in representing the complete data. To ascertain whether the equations generated by the model are efficient in predicting the responses by interaction a non-notable lack of fit is needed. All the *p* values of PS, EE and SI were found to be non-significant and hence the selected model was fit [24]. 123.31, 73.26and 98.91were the model generated F-values for PS,EE and SI, respectively. Therefore,the probability of changes in F-value due to noiseis only 0.01%.

ANOVA resultsoutranged the statistical significance generated by the quadratic equation, also the *p*-value was <0.0500 indicating that the model terms were significant. The experimental design indicated that particle size (PS)was potentially affected by (i) antagonist effect of B[polynomial term] with *p*-value 0.0003 and (ii) synergistic effect of A, B, C and A witha *p*-value of <0.0001, 0.0010, <0.0001 and <0.0001, respectively, being A effects the highest. EE was profoundly contrived by (i) antagonist effect of A,B, AB, BC and B[polynomial term] with *p*-value 0.0003 and (ii) synergism effect of B and A[polynomial term], and among all the significant variables, A[polynomial term] affect theentrapment efficiency (EE)with highenormity. SI was affected significantly by (i) synergistic effect of A, C(*p*-value of <0.0001 and 0.0004); (ii) antagonistic effect of polynomial terms of A, B with *p* values of 0.0024 and 0.0028. Factor A havingthe highest effect stability index (SI). Table 1 and the regression equation confirms the large impact of Zein, chitosan concentrations on the formation of nanocomplex. 

These factors at elevated concentrations resulted in the development of large-sized particles. Because the hydrophobic ingredients affect the internal structure of hydrophobic zein throughout the preparation procedure forming a rigid AV-NC [25]. Adsorption and internal amino-acid repulsions of chitosan molecules at high concentrations may lead to the formation of larger size NC. In general, higher molecular weight chitosan will have a larger spatial distribution size [26]. All these are in accordance with the ANOVA result. On the contrary, ethanol concentration will favor higher EE by preventing the leakage of AV from NC. It is difficult to co-encapsulate the ingredients into the internal cavity of zein nanoparticles, as it is having a very limited internal cavity space, which causes for lowering of EE. SI was affected synergistically by factors A and C. As stated in many literature higher concentrations of factors A and C will favor higher stability. RSM (response surface methodology) was used to predict and determine the independent effect of variables over the individual responses. 3 D RSG (response surface graphs) is vital to elucidate both the interaction and the main effect [Figure 3]. The measured responses are visualized with the help of contour plots. To optimize the sequence of models acquired from study statistical analysis, the global desirability function (D) was applied. Each response was set to limits (SI and EE to maximum and PS to a minimum) to frame an overlay graph to optimize the independent variables. Possible all 3 independent variables were involved in design space for optimization. The independent variables (optimal concentration) indicated a 0.8964 D value (maximum) for both responses in the desirability plot. Therefore, implementing these settings can result in attaining minimum size (169.07 nm) and maximum SI (82.04%), and EE (81.68%). Optimized formulation showed a mean particle size of 169.07 nm closer to the predicted value from the design. By using these optimized concentrations OB-AV-NC as an optimized formulation was prepared and validated the experimental design. Relative error was found to be less than 2%, which confirms the accuracy of the design. 

In general, nanoparticles of zein showed a +ve (positive) charge (32.1 mV), as the pH of the prepared dispersion (4.0) is below the zein iso-electric point (pI-6.5). Zein can be easily fabricated into nanoparticles, but the limited internal cavity space of zein particles may make the process difficult. Hence this problem was solved by forming a structural layer of chitosan around zein nanoparticles and the zein-chitosan nanocomplexes with a unique structure were fabricated using active moieties to form the desired nanocomplex. When the nanocomplexes are further added with chitosan, these coated NC will also highly positive charge and this can be ascribed to the amino group protonation of chitosan [27]. The PDI optimized confirms the monodispersity of the formulation. 

SEM studies confirmed the spheroid shape of AV formulation. This is due to the inherent zein property, which can be self-aggregated into nanosized particles during the preparation of NC [28] [Figure 4]. Several smooth to rough structures were notices and these can be probably generated through the formation of the hydrogen bond between eNH_2_, eOH and eNHCOCH_2_ groups present on the backbone of chitosan.

Figure 5 shows the cumulative percentage of AV permeated from the optimized batch of NC. Results revealed that AV release was incomplete from plain AV (39.28% at end of 168 h) and a biphasic sustained release pattern was observed in the case of AV-NC and AV-LP-EA-NC. Initial burst release (35.23% and34.30%) of AV was observed at the end of 2 h from both the formulations in contrast to plain AV (8.68%). This initial burst release is usually because of entrapped AV near the surface of the nanocomplex. Drug release from the NC can be affected by several parameters such as matrix erosion of nanoparticles [29], rate of water uptake and dissolution/diffusion rate of active moiety. At 8 h, drug release from the formulations reached to plateau, later the drug release followed the sustained pattern, and this can be attributed to the longer diffusion path of deeper entrapped AV. In addition, the hydrophobic nature of zein and chitosan further delays the water penetration resultsintheretardation of AV diffusion. The addition of anti-oxidants indicated no marginal difference in the release of AV from AV-NC and AV-LP-EA-NC formulation.The drug solution was found to be stable even at the end of 168 h, as evident from solution stability data. The drug solution prepared with dissolution medium was stored for a period of 168 h at 5 °C ambient temperature and responses of the stored samples were evaluated, compared to the freshly prepared solution in terms of absorbance, etc. Obtained results were found to be in accordance with the statement. 

AV-NC (nanocomplex formulation without antioxidants), AV-LP-EA (optimized formulation with LP, EA), along plain AV were used to investigate the neuroprotective effect of AV and also to study the impact of antioxidants, using PC12 cells.The effect of different concentrations of glucose (5 mM-NG; 25 mM -HG) on cell viability at 24, 48, 72, 96 h were determined by using MTT assay [Figure 6]. The results showed that HG decreased the cell viability of PC12 cells by 51 % for 72 h in a time-dependent manner (*p* < 0.001). Accordingly, HG was chosen to initiatecell damage and also to assess the neuroprotective effect of stated formulations. 0.012 µM of AV was selected for further studies; this was based on the dose-response and time-dependent studies using different concentrations and time intervals. Pre-treatment of PC12 cells with AV-LP-EA-NC shown the highest cell viability of 92.85% (*p* < 0.001) in comparison with plain AV+ HG and AV-NC. Thus improved viability can be attributed to anti-oxidants (LP, EA) present in the nanocomplex formulation. The mannitol group showed a similar result asthecontrol group. Cell viability of PC12 cells served with AV undergoing NG conditions wasequivalent to the control group. ROS at intracellular levels was measure using DCF-DA and the cells served with HG exhibited maximum limits of ROS succeeding 72 h (*p* < 0.001), a significant reduction in the ROS level was observed with plain AV and also NC formulations. As the intracellular ROS level indirectly measures the antioxidant effect, AV-LP-EA-NC showed maximum reduction (*p* < 0.05). 

Production of ROS is the most primitive response of a cell against the hyperglycaemic condition. ROS generation-long with oxidative stress is the crucial fundamentals interplayed both in the DN pathology and progression. Declined antioxidant ability in cells may lead to a free radical attack on cell components and ROS damage to unsaturated fatty acids results in lipid peroxidation [Figure 7]. As required, the efficiency of AV was enhanced by formulation AV-NC along with efficient antioxidants such as LP and EA. Levels of MDA were measured because it is a sign of 2-fold lipid peroxidation. MDA level was significantly increased on treatment with HG (*p* < 0.001). The treatment with AV and NC formulations noticeably attenuated the change of MDA level. AV-LP-EA-NC showed a maximum reduction than plain AV and AV-NC. MDA and intracellular ROS levels with AV and NC formulations under NG conditions were similar to control and mannitol groups. In general, the cells can protect themselves from damage caused by oxidative stress through their internal antioxidant defense system. Our results were comparable to those in the earlier studies that showed increased ROS level and lipid peroxidation on exposing the PC12 cells to HG. PC12 cells pre-treatment with AV and nanocomplex formulation increased cell viability and inhibited ROS generation and lipid peroxidation [Figure 8]. The probable mechanism for AV anti-oxidant effect is by increasing the cGMP level and this can result in the stabilization of oxidative stress level thus leads to neuron survival. Previous reports also confirmed the antioxidant effect of PDEinhibitors [30]. It is noteworthy that AV-LP-EA-NC showed superior results in contrast with plain AV. This can be credited to the LP and EA. Many reports confirmed the ability of LP andEAaspotentialantioxidants [31]. Finally, PDE-5 inhibitor and nanocomplex formulation with antioxidants can be the potent neuroprotective agents in the clinical treatment of DN [32,33].

## 3. Conclusions

The RSM-based mathematical design using the desirability approach was utilized to systematically improvethe solubility and stability of AV nanocomplex preparation. The formulation containing 2.176 g of chitosan, 7.984 g of zein and 90% *v/v* ethanol concentration had fulfilled the prerequisites of optimum formulation (OB-AV-NC). Optimized formulation was evaluated for various parameters and all the obtained results were in accordance with prerequisites. In-vitro release studies confirmed the enhanced dissolution characteristics (98.15%) and prolonged release of AV (up to 168 h) from the optimized formulation. The prepared formulation showed significant desired neuroprotective activity on PC12 cells even under the HG condition. This favorable effect can be due to its ability to enhance the cGMP level through several pathways. Using natural antioxidants such asLP and EA further enhances the neuroprotective activity of AV in NC formulation. Consequently, PDE-5 inhibitorssuch asAV might be considered as a promising treatment for diabetic neuropathy. The work discussedneedstobe extendedby pre-clinical studies to assess safety in clinical settings.

## 4. Materials and Methods

### 4.1. Materials

MSN Laboratories Hyderabad, Telangana (India), generously gifted AV sample; LP and EA were procured from CDH Pvt. Ltd. Mumbai, Karnataka, India; Zein(95%)and Chitosan (deacetylation degree > 75%, Mol.wt-190,000–310,000 Da) were purchasedfrom Sigma-Aldrich, St. Louis, MO, USA; PC 12 cell linesderived from a transplantable rat pheochromocytoma were gifted by The National Centre for Cell Sciences (NCCS), Pune, Maharashtra, India. All other chemicals and solvents used were of analytical grade. Design Expert Version 12 Stat Ease Inc. (Minneapolis, MN, USA) was used for optimization of proposed formulation. 

### 4.2. Methods

#### 4.2.1. Preparation of Chitosan-Avanafil Nanocomplex

Chitosan avanafil nanocomplex (CS-AV)was prepared by using a modified anti-solvent precipitation method as described by Hu and McClements et al. [34]. Initially, 6–10 g of zein, 0.3% ALA and 0.02% EA were dissolved in 500 mLof ethanol and water mixture by varying concentrations (70–90%) as shown in Table 4. The prepared solution was added to water (1000 mL)carefully using a syringe at 600 rpm, thus zein nanocomplexes were formed spontaneously. 1 g of AV was dissolved in 100 mLof ethanol solution, and this was added to the formed zein nanocomplex at continuous stirring.Finally, the resultant solution was addedwith different concentrations of chitosan solution (Prepared by dissolving 1.5–2.5 g of chitosan in 100 mLof 1M acetic acid). Finally, ethanol was evaporated from the colloidal dispersions using rotary evaporation (40 °C, −0.1 MPa) (R201 L, Shanghai Shengshen, China). The rotary evaporator was used under the vacuum to eliminate all the residues of ethanol. Just before the extract turns dry, water (1–2 mL) was added and then continue to run the extract in the rotary evaporator. As the rotary evaporator allows high vacuum, we can evaporate ethanol at a relatively high rate even at 40 °C.

#### 4.2.2. Optimization of Chitosan-Avanafil Nanocomplex Preparation

The (CS-AV) nanocomplex formulations were optimized statistically by using RSM (Response Surface Methodology). This approach will help in identifying the (a) finest process condition, (b) significant factors and their interactions through fewer experimental runs(Eswari et al. 2016). Selected independent variables were, the concentration of zein(X_1_), ethanol solution (X_2_)and chitosan (X3) at three different levels are coded as −1 (low), 0 (medium) and +1 (high).These factors were optimized for particle size (PS-Y_1_), entrapment efficiency (EE-Y_2_) and stability index (SI-Y_3_). Box–Behnken design was applied using Design Expert 12 (Stat Ease Inc.,Minneapolis, Min, USA), generating 17 experimental trials. Table 1 shows the full experimental plan interns of coded and actual values of selected variables and constraints of dependent variables. Statistical validation of generated polynomial equations was accomplishedby using an analysis of variance (ANOVA). All the experimental runs were applied to different statistical models (such asthe model, 2FI and quadratic, etc.,) and the best-fit model was selected by comparing several statistical parameters such as coefficient of variation (CV), multiple correlation coefficient (R^2^) and adjusted, predicted R^2^values [35]. A quadratic model was used to measure the response in every trial and regression analysis was carried out.

(4) $$Y_{i\left(Quadratic\right)}=b_{0}+b_{1}X_{1}+b_{2}X_{2}+b_{3}X_{3}+b_{4}X_{1}X_{2}+b_{5}X_{1}X_{3}+b_{6}X_{2}X_{3}+b_{7}X_{1}^{2}+b_{8}X_{2}^{2}+b_{9}X_{3}^{2}$$

where *Y_i_*—Dependent variable; *b*_0_—Arithmetic response of experimental trials; *b*_i_—Theestimated coefficient for independent variables *X*_1,_ *X*_2_ and *X*_3_(Main effects)—*X*_1×2_ and *X*_1×3_ and *X*_2_*X*_3_—corresponds to the interaction terms and *X*_1_^2^, *X*_2_^2^ and *X*_3_^2^—The quadratic terms.

### 4.3. Characterization

#### 4.3.1. Particle Size(PS)Determination

The size of the prepared CS-AV nanocomplexeswas evaluated by PCS (Photon correlation spectroscopy) using Malvern Zetasizer (2000, UK), and all the samples were run in triplicate (n = 3) [36,37].

#### 4.3.2. Entrapment Efficiency(EE)Determination

Prepared CS-AV nanocomplex formulations were weighed and freeze-dried (EBT 12 N. Esquire Biotech, India) in a petri dish. The required amount of solvent was added to the weighed sample, mixed and then the sample was centrifuged at 10,000 RPM (REMI centrifuge, C-24 BL, India) for 1 h [38]. Further,the washingwas continued, all the supernatants and washings were collected and later dried on a water bath. Methanol was added to the above-dried extract and diluted. AV peak area was measured at 230 nm for AV using earlier reported HPLC (High-performance liquid chromatography)method [39]. EE was calculated using the following equation,

(5) $$\mathrm{EE}\left(\%\right)=\frac{\mathrm{Ctotal}-\mathrm{Cfree}}{\mathrm{Ctotal}}$$

C_total_— Theoretical concentration

C_free_— Free amount of drug detected in supernatant and washings.

#### 4.3.3. Stability Index(SI)

The optimum formulations were filled into the amber-coloredscrew cap glass bottles and stored at 4.0 ± 1 °C; 25 ± 2 °C as per ICH Q1A (R2) guidelines for about 30 days in a stability chamber (CHM-6S, Remi Electro-Tech Ltd., India). At predefined intervals of time, samples were aliquoted and evaluated for size, drug content and compared with initial values [40]. 

#### 4.3.4. The Rationale of Experimental Design

An optimized batch of nanocomplex (OB-AV-NC) was prepared by utilizingtheoptimum concentration of independent factorsindicated by softwareandevaluated [41]. The optimized result of the experimental design can be validated by calculating relative error by comparing the predicted responses with practical responses as given in Equation (2).Plain AV-NC (nanocomplex formulation without antioxidants) and AV-LP-EA (optimized formulation with LP, EA) were prepared for further studies.

(6) $$\mathrm{Relative}\;\mathrm{error}\left(\%\right)=\frac{\mathrm{Predicted}\;\mathrm{value}-\mathrm{Practical}\;\mathrm{value}}{\mathrm{Predicted}\;\mathrm{value}}\times 100$$

#### 4.3.5. Polydispersity Index(PDI) and Zeta Potential(mV) Determination

The sample wasdiluted with deionized distilled water (1:10) in the capillary cell at 25 °C.Both PDI and surface charge were measured for nanocomplex formulations using Malvern Zetasizer (2000, UK) [42].

#### 4.3.6. Scanning Electron Microscopy(SEM)

SEM was used to investigate the surface morphology and shape of the prepared nanocomplex formulation. Samples were mounted on a stub and sputter-coated with gold and imaged using SEM (JEOL, JSM-6100, Tokyo, Japan). 

#### 4.3.7. Differential Scanning Calorimetry(DSC) Studies

DSC studies were performed for pure AV and optimized formulation using DSC60 Shimadzu Corporation, Japan. Calibrations of temperature were performed by using Indium as a standard. Sealed and perforated aluminum pans were used, and DSC scans were performed by a programmed thermal analyzer system [43]. Prepared samples were run at a scanning speed of 10 °C/min from 50 to 350 °C.

#### 4.3.8. In-Vitro Drug Release Studies

To determine the cumulative release of AV from prepared nanocomplex, an in-vitrorelease study using Franz diffusion cell was performed. Different formulations, AV-NC, AV-LP-EA and plain AVwere placed in the respective donor chamber; to pass through the dialysis membrane (cut-off molecular weight of 12 kDa). Phosphate buffer (pH 7.2) was used as a releasemedium and samples were collected at predetermined intervals. The aliquoted sampleswere quantified for AV using the previously reported high-performance liquid chromatography (HPLC) method [44].The withdrawn samples were dissolved in 10 mL acetonitrile. After filtration through a 0.45-μm membrane filter, the filtrate was diluted to 1:100 with acetonitrile and the drug content was determined by HPLC using acetonitrile:water (90:10) (*v/v*) as the mobile phase, at a flow rate of 1 mL/min (LC-10 AD isocratic pump; Shimadzu, Japan). The HPLC system included a reversed-phase C18 column (3.9 mm × 300 mm, particle size 4 μm; Waters, USA) and aspectrofluorimetric detector at a wavelength of 238 nm (RF-551; Shimadzu, Japan).

#### 4.3.9. Cell Culture and Treatment

PC12 cells were grown in DMEM (Dulbecco’s modified eagles’ medium) and the medium was supplemented by a 1% antibiotic mixture (penicillin-streptomycin), 10% fetal bovine serum at humidified atmosphere (5% CO_2_ at 37 °C). Cells were treated with nerve growth factor (NGF) at 50 ng/mL,everyalternate day for about 6 days. 5 mM, 25 mM of D-glucose in Dulbecco’s Modified Eagle Medium (DMEM) were identified as control and hyperglycemic (HG) medium. The cells were incubated for different time intervals (24, 48, 72 and 96 h) to know the approximate incubate time. The control group was supplemented with D-Mannitol (3.5 g/L), which acts as an osmotic control 45](. Dimethyl sulfoxide (DMSO) (0.5%) was used to prepare the AV drug solution. AV concentration was selected on basis of dose-response and time course study with different concentrations such as 0.0001, 0.004, 0.008, 0.012, 0.05, 0.1 and 1 µM(data not included).Treatment groups includedare(i) plain AV (AV was incubated with the cells for 60 min), (ii) AV + glucose (AV was incubated for 60 min and then exposes to HG medium for 72h) (iii)AV-NC (AV nanocomplex without antioxidants) and (iv)AV-LP-EA-NC (AV nanocomplex with antioxidants).

#### 4.3.10. Cell Viability Measurement

MTT{3-[4,5-dimethylthiazol-2-yl]-2,5-diphenyltetrazolium bromide} reduction assay was used to determine cell viability. Dark blue-coloredformazan crystals were solubilized in DMSO and added to the intact cells. Finally, absorbance was measured at 550 nmusing a Microplate reader (Bio Tek Instruments, Weinowski, VT, USA) [45,46]. Obtained results were expressed as the percentage of MTT reduced in comparison to the control group cells.

#### 4.3.11. Intracellular ROS Measurement

DCF-DA (2′, 7′- dichlorofluoresceindiacetate) was used to determine the intracellular accumulation of oxygen reactive species (ROS). Cell suspension (1 × 10^6^/mL) was exposed to 10 μM of DCF-DA and then incubated at 37 °C for 1 h. Consequently,thecells were washed usingaphosphate buffer solution (Ph7.2). The fluorescence of the cells was measured by using a Microplate reader (Bio Tek Instruments, Weinowski, VT, USA) at 485 and 528 nm for excitation and emission, respectively [47]. 

#### 4.3.12. Lipid Peroxidation Measurement

Malondialdehyde (MDA) reaction with thiobarbituric acid (TBA), which generates purple color, can be measured by a double heating method [47]. For a short time, cell lysate was mixed with 10% *w/v* of the trichloroacetic acid solution, then boiled in a water bath for about 15–20 min, and then samples were centrifuged (3000 rpm for 10 min). The supernatant of all the samples was collected and then transferred to 0.67% *w/v* TBA solution contained tube. Then, the tubes were kept in hot water for about 15 min, and then absorbance was measured in a Microplate reader (Bio Tek Instruments, Weinowski, VT, USA) at 532 nm in contrast with blank.

## Figures and Tables

**Table 1:** Projected trial batches and their responses for Box-Behnken design.

Run	A:ZeinConcentration (g)	B:EthanolConcentration (% *v*/*v*)	C:Chitosan (g)	PS (nm)	EE (%)	SI (%)
1	10	80	2.5	355	51	98
2	10	90	2	315	67	95
3	6	80	2.5	199	72.5	69
4	8	80	2	179	70	83
5	8	80	2	170	67	85
6	10	80	1.5	286	52	92
7	8	90	1.5	132	76	78
8	6	90	2	158	87	59
9	8	80	2	172	68	84
10	8	70	2.5	172	57	82
11	10	70	2	258	46	95
12	8	80	2	169	68	86
13	8	70	1.5	109	74	74
14	8	80	2	165	66	84
15	8	90	2.5	185	84.5	82.5
16	6	70	2	125	86	58
17	6	80	1.5	148	82	57

**Table 2:** Fit Summary for Responses.

	PS		EE		SI	
Source	Adjusted R^2^	Predicted R^2^	Adjusted R^2^	Predicted R^2^	Adjusted R^2^	Predicted R^2^
Linear	0.6078	0.3678	0.7534	0.5873	0.9167	0.8942
2FI	0.4954	-0.4754	0.8654	0.5955	0.8992	0.8251
Quadratic	0.9857	0.9199	0.9760	0.8845	0.9822	0.9035
Cubic	0.9944	---	0.9854	---	0.9921	---

**Table 3:** Estimated effects for the different factors in preparing AV-NC.

	PS		EE		SI	
Source	F-Value	*p*-Value	F-Value	*p*-Value	F-Value	*p*-Value
Model	123.31	<0.0001*	73.26	<0.0001*	98.91	<0.0001*
A-Zein concentration	628.92	<0.0001	428.91	<0.0001	795.78	<0.0001
B-Ethanol concentration	29.28	0.0010	91.50	<0.0001	1.28	0.2947
C-Chitosan concentration	102.71	<0.0001	12.45	0.0096	39.44	0.0004
AB	2.12	0.1883	27.60	0.0012	0.0848	0.7793
AC	1.19	0.3105	4.99	0.0607	3.05	0.1241
BC	0.3688	0.5628	44.87	0.0003	1.04	0.3420
A^2^	306.54	<0.0001	6.69	0.0361	21.58	0.0024
B^2^	46.12	0.0003	43.24	0.0003	20.22	0.0028
C^2^	2.05	0.1949	1.22	0.3057	3.27	0.1136
Residual						
Lack of Fit	4.64	0.0863**	2.51	0.1976**	3.96	0.1085**

* Significant Model. ** Non-significant lack of fit.

**Table 4:** Full experimental plan interns of coded and actual values of selected variables and constraints of dependent variables for Box–Behnken design.

Factors/Independent Variables	Levels			Responses/Dependent Variables	Constraints
	−1	0	+1		
Concentration of Zein (g)-X_1_	6	8	10	Particle size (nm)	Minimum
Ethanol solution concentration (%*v/v*)- X_2_	70	80	90	Entrapment Efficiency (%)	Maximum
Concentration of Chitosan (g)-X_3_	1.5	2	2.5	Stability Index (%)	Maximum

## Data Availability

Not applicable.

## Supplementary Materials

The following are available online at https://www.mdpi.com/article/10.3390/gels7030096/s1.
