# Supplementary figures and images for: Preparation, Optimization and Evaluation of Chitosan-Based Avanafil Nanocomplex Utilizing Antioxidants for Enhanced Neuroprotective Effect on PC12 Cells
Source: Gels. 2021 Jul 16;7(3):96. doi: 10.3390/gels7030096 (PMC8293062; doi:10.3390/gels7030096)

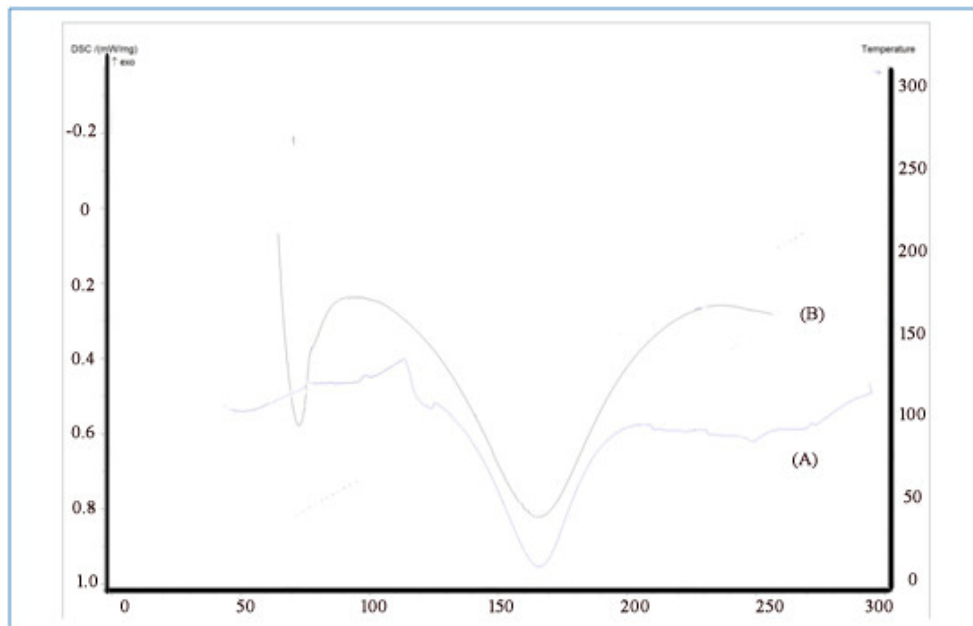

DSC thermograms of (A) pure AV and (B) optimized formulation.

Supplement: Supplementary file 1 [file gels-07-00096-s001.zip › gels-1237703-supplementary.pdf]
